# Visualization of Viral Infection Dynamics in a Unicellular Eukaryote and Quantification of Viral Production Using Virus Fluorescence *in situ* Hybridization

**DOI:** 10.3389/fmicb.2020.01559

**Published:** 2020-07-17

**Authors:** Yaiza M. Castillo, Marta Sebastián, Irene Forn, Nigel Grimsley, Sheree Yau, Cristina Moraru, Dolors Vaqué

**Affiliations:** ^1^ Department of Marine Biology and Oceanography, Institute of Marine Sciences (CSIC), Barcelona, Spain; ^2^ Institute of Oceanography and Global Change (IOCAG), University of Las Palmas de Gran Canaria (ULPGC), Las Palmas de Gran Canaria, Spain; ^3^ Integrative Biology of Marine Organisms (BIOM), Sorbonne University, CNRS, Oceanographic Observatory of Banyuls, Banyuls-sur-Mer, France; ^4^ Department of the Biology of Geological Processes, Institute for Chemistry and Biology of the Marine Environment, Oldenburg, Germany

**Keywords:** virus fluorescence *in situ* hybridization, *Ostreococcus tauri*, *Ostreococcus tauri* virus 5, virus-host interactions, culture system, marine picoeukaryote

## Abstract

One of the major challenges in viral ecology is to assess the impact of viruses in controlling the abundance of specific hosts in the environment. To this end, techniques that enable the detection and quantification of virus-host interactions at the single-cell level are essential. With this goal in mind, we implemented virus fluorescence *in situ* hybridization (VirusFISH) using as a model the marine picoeukaryote *Ostreococcus tauri* and its virus *Ostreococcus tauri* virus 5 (OtV5). VirusFISH allowed the visualization and quantification of the proportion of infected cells during an infection cycle in experimental conditions. We were also able to quantify the abundance of free viruses released during cell lysis, discriminating OtV5 from other mid-level fluorescence phages in our non-axenic infected culture that were not easily distinguishable with flow cytometry. Our results showed that although the major lysis of the culture occurred between 24 and 48 h after OtV5 inoculation, some new viruses were already produced between 8 and 24 h. With this work, we demonstrate that VirusFISH is a promising technique to study specific virus-host interactions in non-axenic cultures and establish a framework for its application in complex natural communities.

## Introduction

Marine viruses have been studied during the last 3 decades mostly using microscopy (Noble and Fuhrman, 1998) and flow cytometry (FCM; Marie et al., 1999) for the enumeration and estimation of viral production. However, in the last few years, the development of high throughput sequencing techniques has considerably changed the field, and our knowledge about viral communities has exponentially increased. These new sequencing approaches provide information about the viral taxonomic and genomic diversity, about their biogeography and, to a certain extent, about their potential hosts (e.g., Chow et al., 2015; Labonté et al., 2015; Castillo et al., 2019). However, they do not allow the visualization of specific virus-host interactions and the monitoring of infection dynamics, which are crucial to better understand the role of viruses in shaping microbial communities and biogeochemical cycles.

Attempts to identify virus-host associations date back to the 1990s, when the role of viruses in the marine environment started to be recognized. Hennes et al. (1995) were pioneers in using fluorescently stained virus isolates to identify and enumerate their hosts in natural communities. Years after, Tadmor et al. (2011) used microfluidic digital PCR to detect specific phage-host associations in the termite gut. With this method, they managed to directly detect the phage-host association by targeting genes from both components without culturing but with no visual representation of the infection.

A few years ago, Allers et al. (2013) developed phage fluorescence *in situ* hybridization (phageFISH) and used it to monitor phage infections at the single-cell level in a marine podovirus-gammaproteobacterial host system. PhageFISH uses mixtures of polynucleotide probes labeled with digoxigenin to target phage genes, and a single horseradish peroxidase (HRP) labeled oligonucleotide probe to target host rRNA. The signal from the two types of probes is amplified and visualized by catalyzed reporter deposition (CARD) of fluorescently labeled tyramides. Compared to the method from Hennes et al. (1995), where the infection was forced by adding stained viruses to identify the host within natural communities, phageFISH enables the visualization of the interaction of specific virus-host pairs, because it simultaneously targets the virus and the host. More recently developed, direct-geneFISH (Barrero-Canosa et al., 2017) uses simultaneously a mixture of polynucleotide probes directly labeled with fluorochromes, to detect specific genes, and a single oligonucleotide probe, carrying multiple fluorochromes, to identify bacterial cells.

In the present work, we combined the phageFISH and direct-geneFISH techniques to develop VirusFISH with the aim of allowing (i) the identification and quantification of specific virus-unicellular eukaryote interactions at the single-cell level and (ii) the identification and quantification of free virus particles. Our procedure involves two different steps. First, a CARD-FISH step is used to detect host cells, with HRP-labeled oligonucleotide probes targeting the 18S rRNA. Then, a gene-FISH step is applied to detect viruses, using multiple polynucleotide probes labeled with fluorochromes that target specific viral genes. Since this last step can be used to detect both intracellular viruses and free viral particles, we named it VirusFISH. To visualize the host-virus interaction, VirusFISH needs to be combined with the CARD-FISH step mentioned above.

As proof of principle, we used VirusFISH to monitor viral infections in a culture of the unicellular green alga *Ostreococcus tauri* with the virus *O. tauri* virus 5 (OtV5).

## Materials and Methods

### Host-Virus System

*O. tauri*, from the Mamiellophyceae class, is an important member of the eukaryotic photosynthetic picoplankton in coastal and open sea environments (Countway and Caron, 2006; Massana, 2011). It is the smallest free-living eukaryote known (~1 μm), capable of rapid growth in culture (Countway and Caron, 2006), and it has an extremely simplified cellular organization (Chrétiennot-Dinet et al., 1995).

In order to develop VirusFISH, we chose the *O. tauri* RCC4221 (Roscoff Culture Collection, NCBI accession number txid70448) – OtV5 (NCBI accession number EU304328) system. OtV5 is a member of the Phycodnaviridae family that was isolated from the Bages lagoon in 2006 (Derelle et al., 2008). It is a fast-lytic icosahedral dsDNA virus with a 186,234 bp genome and a capsid diameter of ~120 ± 30 nm. The infection dynamics of this virus-host system has already been described (Derelle et al., 2008).

### OtV5 Probe Design and Synthesis

For the detection of the OtV5 virus, we designed 11 dsDNA polynucleotide probes (300 bp each) using the software geneProber web service (http://gene-prober.icbm.de/, see Supplementary Material section for a step-by-step description of the whole procedure). These 11 probes covered a total of 3,998 bp of the OtV5 viral genome (Supplementary Table S1), offering sufficient sensitivity to detect single genes (Barrero-Canosa et al., 2017), and thus single viruses. Each probe synthesis was done by obtaining the corresponding polynucleotides by PCR, and then all probes were mixed and labeled with the Alexa594 fluorochrome, following Barrero-Canosa et al. (2017). The PCR was set up as follows: 10 pg of OtV5 DNA was added to a reaction mixture containing 200 μM (each) deoxyribonucleoside triphosphates (Invitrogen, USA), 1 μM of each primer, 1x PCR buffer (Invitrogen), and 5 U of *Taq* DNA polymerase (Invitrogen). The amplification was performed in a C1000TM Thermal Cycler (Bio-Rad) with an initial denaturation step at 95°C (5 min), followed by 30 rounds at 95°C (1 min), X°C (30 s), and 72°C (30 s), and a final extension at 72°C (10 min). X value corresponds to the optimal annealing temperature for each of the primers determined after performing gradient PCRs. All OtV5 primers had an optimal annealing temperature of 62.5°C, with the exception of primers #3 and #5 that had an annealing temperature of 65.5°C. For each polynucleotide, several PCRs were done to obtain a minimum of 400 μl PCR volume. This volume was purified on a single purification column using the QIAquick PCR purification kit (Qiagen, Germany, cat.no. 28106) and resuspended in a TE solution (5 mM Tris-HCl and 1 mM EDTA, pH 8.0). The polynucleotide length was checked by agarose gel electrophoresis, and the concentration was measured spectrophotometrically using a NanoDrop 1000 (Fisher Thermo Scientific). Then, all 11 polynucleotides were mixed equimolarly to yield a total of 1 μg DNA in 10 μl TE. Later, the probe mixture was heated to 95°C for 5 min to denature it and then incubated for 30 min at 80°C with 10 μl of the Alexa594 dye for the fluorescent labeling (Ulysis™ Alexa Fluor® 594 Nucleic Acid Labeling Kit, Thermofisher, MA, USA, cat.no: U21654). The unbound Alexa594 was removed using chromatography columns (Micro Bio-spin chromatography columns P-30, Bio-Rad, California, USA, cat.no. 732-6202). The concentration of the probe mixture and the labeling efficiency with the Alexa594 were determined spectrophotometrically using a NanoDrop 1000 with the multi-array option and N-50. For a successful detection of the virus, we observed that the average labeling efficiency should be at least six Alexa594 dyes per probe (see further details in the step-by-step protocol in the Supplementary Material section). Fluorescent probes were stored at −20°C until use.

### Experimental Viral Infection of *O. tauri*

The host strain *O. tauri* RCC4221 was grown in 60 ml of L1 medium (Guillard and Hargraves, 1993) in aerated flasks (Sarstedt) and incubated at 21.5°C (±0.5°C) with white light ~100 μE and a 10:14 h photoperiod (light:darkness), until stationary phase [7.16 × 10^7^ ± 3.57 × 10^6^ cells ml^−1^, estimated by 4′-6-diamidino-2-phenylindole (DAPI) counts (Porter and Feig, 1980), for better detection of individual cells]. Triplicate *O. tauri* cultures (20 ml) were infected at 12 PM with 1 ml of OtV5 inoculum (1.3 × 10^7^ ± 4.3 × 10^6^ viruses ml^−1^, estimated by plaque-forming units), resulting in a 0.01 multiplicity of infection (MOI). Non-infected triplicate *O. tauri* cultures (inoculated with 1 ml of L1 medium) were used as control. After OtV5 inoculation, samples (900 μl) were taken over 3 days at times 0, 8, 24, 48, and 72 h, and fixed with 100 μl of freshly filtered formaldehyde (3.7% final concentration) for 15 min at room temperature. Then, 500 μl of fixed sample + 5 ml 30 KDa filtered sea water was filtered through 25 mm 0.2 μm pore size polycarbonate white filters (Merck™ GTTP02500) to retain cells (dilution with 30 KDa filtered sea water helps to have a homogenous distribution of cells on the filter during the filtration process without altering the sample composition). The 0.2 μm pore size polycarbonate filters were embedded in 0.1% (w v^−1^) low gelling point agarose to prevent cell loss, and treated for 1 h with 96% ethanol and 1 h with pure methanol, to remove cellular pigments that can interfere with the CARD-FISH signal (Supplementary Figure S1), and 10 min with HCl to inactivate endogenous peroxidases (Pavlekovic et al., 2009). All filters were kept at −20°C until hybridization. For the detection of free viruses, the sample was filtered through a 25 mm 0.2 μm pore size syringe filter to remove cell debris, 500 μl subsamples were fixed with formaldehyde (3.7% final concentration) for 15 min at room temperature, and viral particles were collected onto 25 mm 0.02 μm pore size anodisc filters (Whatman®).

### Detection of *O. tauri* Cells Using 18S rRNA Targeted CARD-FISH

*O. tauri* cells were labeled using Catalyzed Reporter Deposition-FISH (CARD)-FISH following Pernice et al. (2015), with the 18S rRNA *Ostreococcus* spp. specific probe OSTREO01 (Not et al., 2004). Briefly, the hybridization was carried out by covering filter pieces with 20 μl of hybridization buffer (HB) containing 40% deionized formamide and incubating at 35°C overnight (see Supplementary Material section for details on the HB composition). After two successive washing steps of 10 min at 37°C in a washing buffer, and a equilibration in phosphate-buffered saline for 15 min at room temperature (Cabello et al., 2016), the signal was amplified for 1 h at 46°C with Alexa488-labeled tyramide. Filters were then placed in phosphate-buffered saline twice for 10 min, rinsed with MilliQ water and air-dried.

### Detection of Intracellular and Free OtV5 Viruses

OtV5 viruses were labeled using a modified version of the direct-geneFISH protocol (Barrero-Canosa et al., 2017). OtV5 associated to *Ostreococcus* cells were visualized on 0.2 μm pore size filters that had been previously hybridized with the *Ostreococcus* CARD-FISH probes. Free OtV5 particles (~120 nm in diameter) produced during the experiment were monitored on 0.02 μm pore size filters. The hybridization was done by covering the filter pieces with 25 μl of 40% formamide hybridization buffer (see composition in the step-by-step protocol in the Supplementary Material section) containing the OtV5 probes and incubating first for 40 min at 85°C, and then for 2 h at 46°C. The volume of probe mixture labeled with Alexa594 to add to the HB was calculated based on the following formula, according to Barrero-Canosa et al. (2017):

$$\begin{array}{l} \;\;\;\;\;\;\;\;\left(25\mu l\;\mathrm{HB}•\;nu\mathrm{mber of filters}\right)\;•\\ \;\;\;\;\;\;\;\;\left(\frac{62\mathrm{pg}}{\mu l}\;\mathrm{final probe concentration}\;•\;\mathrm{number of total probes}\right)/\\ \;\;\;\;\;\;\;\mathrm{Viral probe concentration}\;\left(\frac{\mathrm{ng}}{\mu l}\right)\;•\;1000=\mu l\;\mathrm{probe mixture} \end{array}$$

The formula above assumes that the volume of HB for each filter portion is 25 μl and 62 pg·μl^−1^ is the desired final concentration for each polynucleotide, according to Barrero-Canosa et al. (2017).

Finally, samples were washed at 48°C for 15 min with gentle shaking in a washing buffer (see composition in the step-by-step protocol in the Supplementary Material section), rinsed with MilliQ water and air-dried.

### Sample Mounting, Visualization, and Image Analysis

The CARD-FISH signal for this tiny picoeukaryote is not very strong, because it is limited to its small cytoplasm. Thus, after hybridization, the 0.2 μm filters were counterstained with DAPI at 0.5 μgml^−1^ to facilitate the counting of *O. tauri* cells that appear clustered. Filters were then mounted in an antifading reagent (77% glycerol, 15% VECTASHIELD, and 8% 20x PBS; Cabello et al., 2016). Images were manually acquired using a Zeiss Axio Imager Z2m epifluorescence microscope (Carl Zeiss, Germany) connected to a Zeiss camera (AxioCamHR, Carl Zeiss MicroImaging, S.L., Barcelona, Spain) at 1000x magnification through the AxioVision 4.8 software. The DAPI signal from *O. tauri* was observed with the specific UV filter set (370/40 nm excitation, 425/46 emission, and FT 395 beam splitter), while the 18S rRNA CARD-FISH signal from *O. tauri* was observed using a filter set specific for Alexa488 (475/30 nm excitation, 527/54 BP emission, and FT 495 beam splitter). OtV5 was observed using a filter set specific for Alexa594 (585/35 nm excitation, 615 LP emission, and FT 570 beam splitter). All pictures were taken using the same intensities and exposure times (400 ms for the *O. tauri* and 1 s for the virus detection).

Total free viruses (i.e., both OtV5 and phages present in the non-axenic culture), collected on the 0.02 μm pore size anodisc filters, were counterstained with SYBR Gold (SYBR™ Gold solution, Invitrogen) at 2x final concentration for 12 min and then rinsed abundantly with MilliQ water to remove excess stain. Filters were finally mounted on slides with an antifading mounting solution (CitiFluor™ Glycerol-PBS Solution AF1). Images were acquired on the same Zeiss microscope and camera at 1000x magnification. OtV5 were observed by epifluorescence microscopy under 585/35 nm excitation, 615 LP emission wave-lengths, and FT 570 beam splitter, using 1 s of exposure time, and total viruses (OtV5 and phages) under 475/30 nm excitation, 527/54 BP emission wave-lengths, and FT 495 beam splitter, using 50 ms of exposure time. Due to the long exposure times needed to visualize the OtV5 viruses there was some non-specific Alexa594 signal (red color) in the micrographs, but co-localization with SYBR Gold (green color) resulted in a drastic reduction of this unspecificity (Supplementary Figure S2). Thus, only the VirusFISH red signal that overlapped with a SYBR Gold green fluorescence signal, was considered a true OtV5 particle. All pictures were taken using the same intensities and exposure times mentioned above. Image analysis for free virus detection was done using the software ACMEtool 3 (July 2014; M Zeder, Technobiology GmbH, Buchrain, Switzerland).

**Figure 1:**
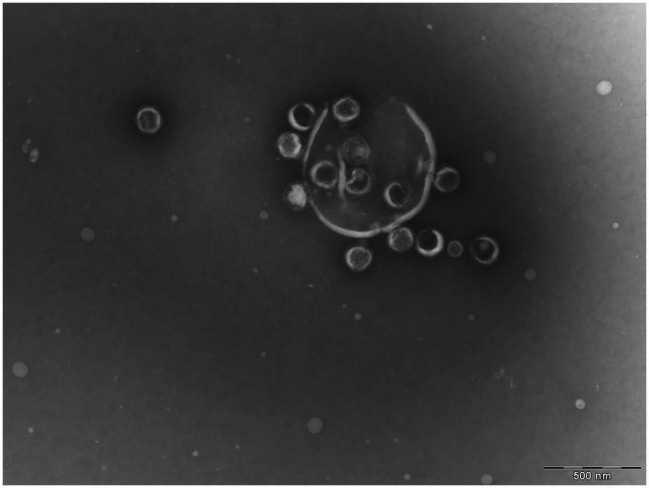
Transmission electronic microscope image of a lysed *Ostreococcus tauri* cell. Released *Ostreococcus tauri* virus 5 (OtV5) virions are observed surrounding cell debris.

During the image analysis, we observed that a fraction of the OtV5 virions released from the cells during lysis was trapped on an extracellular organic matrix surrounding cell debris (here referred to as viral clouds, Figure 1; Supplementary Figure S3) and retained on the 0.2 μm filters. Thus, for 48 and 72 h, when most cells were lysed, we also calculated the abundance of OtV5 retained on the 0.2 μm filters. To this end, for those timepoints, 10 images of each of the triplicate cultures were analyzed using AcmeTool 3, and the area of the viral clouds around the cells was measured. The number of virus trapped in the organic matrix was obtained by dividing the total area of viral clouds in the 10 images by the average area of an OtV5 virus, obtained from the 0.02 μm filters (at 48 h, *n* = 2,432 viral clouds areas; at 72 h, *n* = 307 viral clouds areas; and free OtV5, *n* = 30,000 OtV5 particles areas). The total OtV5 production at 48 and 72 h, in each of the triplicate cultures, was estimated as the sum of the free virus abundance on the 0.02 μm filters plus the viral abundance retained on the 0.2 μm filters. Despite this effort, we acknowledge that viral production calculated this way could be underestimated, as it is possible that some viral particles on the 0.2 μm filters may be hidden behind others and not displayed on a single layer.

### Comparison of OtV5 Counts With VirusFISH and Flow Cytometry

Since flow cytometry is usually used to quantify viral production during infection experiments, we compared OtV5 counts obtained with flow cytometry and VirusFISH. Triplicate 500 μl samples were taken from a healthy and an infected *O. tauri* culture 96 h post OtV5 inoculation. Samples were filtered through a 25 mm 0.2 μm pore size syringe filter to remove cells and cell debris. For VirusFISH, 500 µl of the 0.2 µm filtered subsamples were fixed with formaldehyde (3.7% final concentration) for 15 min at room temperature, and the viral particles were collected onto 25 mm 0.02 μm pore size anodisc filters (Whatman®) and kept at −20°C. Later, the VirusFISH protocol was applied as explained above.

For the flow cytometry counts, the 0.2 μm filtered samples were fixed with glutaraldehyde (0.5% final concentration) for 30 min at 4°C and counted in a FACSCalibur (Becton & Dickinson) flow cytometer following Brussaard (2004). Briefly, samples were diluted 1/1000 from the infected culture, and 1/100 from the control, using TE-buffer (10:1 mM Tris:EDTA). Then, samples were stained with SYBR Green I (5 μl in 500 μl diluted sample), incubated at 80°C for 10 min, attemperated 5 min in the dark, and ran at a medium flow speed with a flow rate of 64 μl min^−1^. Counts were made for 60 s using the following settings: SSC 625, FL1 530. In the cytograms low-level, mid-level, and high-level fluorescence viruses were distinguished as in Brussaard et al. (2010).

### Viral Morphologic Characterization by Transmission Electron Microscopy

The viral lysate of *O. tauri* was prepared for transmission electron microscopy (TEM) at the Unitat de Criomicroscopia Electrònica (Centres Científics i Tecnològics, Universitat de Barcelona). For this, 6 μl of the viral lysate was placed onto a parafilm with a fresh glow-discharged coated carbon grid on them for 1 min. The adsorbed viruses in the grid were negatively stained by adding five drops of uranyl acetate solution (2%, final conc) for 10 s. Excess stain was drawn off with filter paper and the grid was air-dried. The grids were observed in a Jeol 1010 (Jeol, Japan) transmission electron microscope operating at 80 kv equipped with a CCD camera SIS Megaview III and AnalySIS software.

## Results

### The OtV5 – *O. tauri* Infection Dynamics as Revealed by VirusFISH

**Figure 2:**
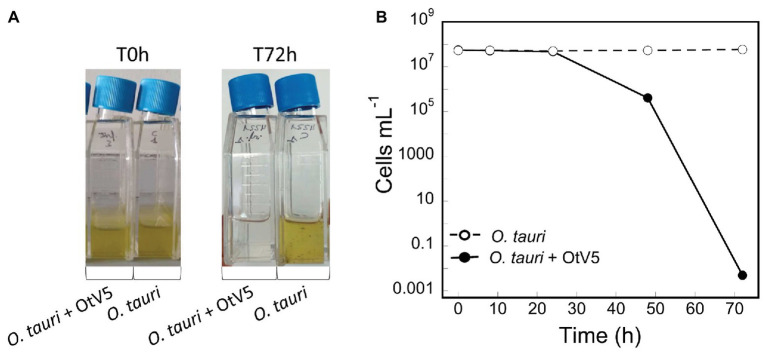
Dynamics of the infection of *O. tauri* with OtV5. **(A)** Infection and control culture flasks at time 0 and 72 h. **(B)** *O. tauri* cell abundances (average ± standard error) detected by catalyzed reporter deposition fluorescence *in situ* hybridization and counted by epifluorescence microscopy in both the infected (solid circles) and the control (empty circles) triplicate cultures.

**Figure 3:**
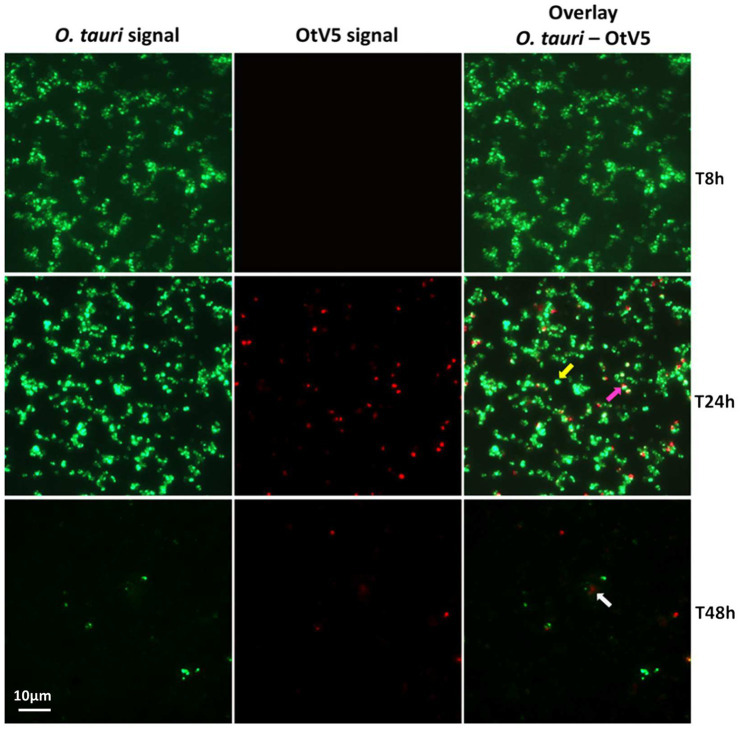
Micrographs of the evolution of the infection from time 8 to 48 h. Left: *O. tauri* only. Centre: OtV5 only. Right column: overlay of *O. tauri* host cells in green (Alexa488) and virus in red (Alexa594). Yellow arrow: non-infected *O. tauri*, pink arrow: infected *O tauri*, gray arrow: cloud of viruses retained on the filter by the organic matter released during the lysis. See supplementary Figures for a complete temporal overview of both the infection and the control cultures.

A non-axenic culture of *O. tauri* was infected with the virus OtV5, at a MOI of 0.01 and an uninfected culture was grown in parallel, as a control (Figure 2A). Using 18S CARD-FISH combined with VirusFISH, the two cultures were followed for 72 h, quantifying (i) the absolute abundance of *O. tauri* cells and (ii) the relative and absolute abundance of infected *O. tauri* cells. The infected culture experienced a dramatic decrease in cell density, going from 4.8·10^7^ to 4.1·10^5^ cells ml^−1^ in 48 h (Figures 2B, 3; Supplementary Figure S4). At 72 h, almost no *O. tauri* cells were detected (Figure 2B; Supplementary Figure S4), consistent with the clearing of the infected culture (Figure 2A). The same infection dynamics were observed in three previous *O. tauri*- OtV5 experiments (data not shown). The absence of any red signal (i.e., fluorescence of the OtV5 probes) in the control treatment (Supplementary Figure S5) confirms the lack of false positives (i.e., non-specific binding of the OtV5 probes to host cells) during the hybridization.

**Figure 4:**
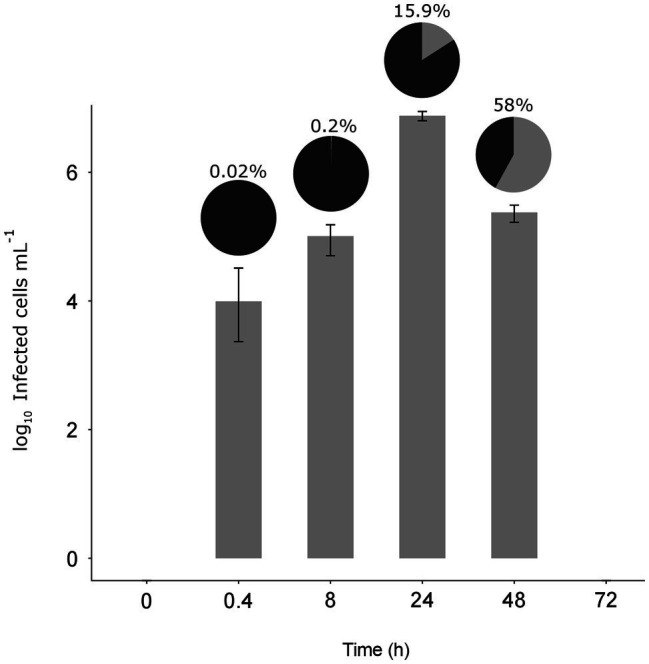
Dynamics of the infected cells. Bar plot shows the number of infected *O. tauri* at each time (± standard error of the three replicates). Pie charts on top of each bar show the percentage of infected cells (gray) with respect to the total *O. tauri* abundance.

At the MOI used, rapid adsorption of all the viral particles added would theoretically result in 1% of infected cells (considering “infected” cells those with either viruses adsorbed or actual infections). However, despite infected cells being visible as early as 0.4 h, their abundance was very low at both 0.4 and 8 h (0.02 and 0.2%, respectively), suggesting that not all viral particles had yet been adsorbed (Figure 4). Nevertheless, the fact that at 24 h, we found 16% of the population infected implies that between 8 and 24 h there had been some production of viruses that had gone on to infect more cells in the culture. Later, at 48 h, the abundance of cells decreased by two orders of magnitude and 60% of the remaining cells were infected (Figures 3, 4). In contrast, the abundance of *O. tauri* cells in the control cultures remained relatively constant along the experiment and, as expected, no infected cells were observed (Figure 2B; Supplementary Figure S5).

### Dynamics and Abundances of Free OtV5 Particles

**Figure 5:**
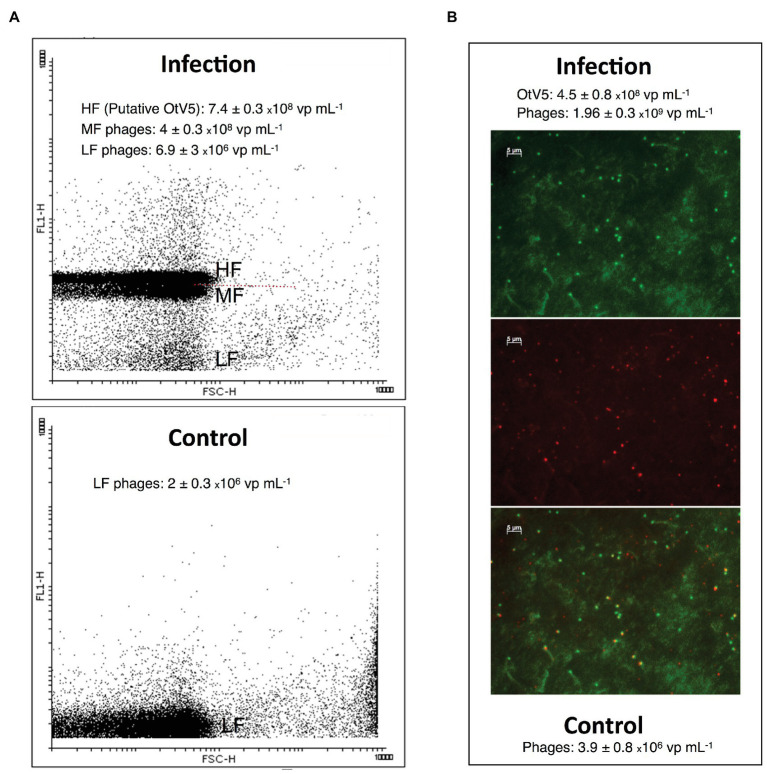
Comparison of free virus counts using flow cytometry (FCM) and virus fluorescence *in situ* hybridization (VirusFISH). **(A)** Cytogram of viral events from an infected (96 h after OtV5 inoculation, upper panel) and a healthy *O. tauri* culture (Control, lower panel). The cytogram of the infected culture shows mid-level (MF) and high-level (HF) fluorescence viruses (presumably phages and putative OtV5), and also low-level fluorescence viruses (LF). The dashed red line separate MF from HF virus. Only LF viruses (phages) are seen in the control. **(B)** Micrographs of the 0.02 μm filter of an infected culture. Top: total viruses stained with SYBR Gold. Center: VirusFISH labeled OtV5 viruses. Bottom: overlay of SYBR Gold and VirusFISH signals for OtV5 viruses. Values in the graphs represent the values obtained with VirusFISH (on the 0.02 μm filter) and flow cytometry for the same 0.2 μm prefiltered samples for both the infected and the healthy *O. tauri* culture.

We also used VirusFISH for the detection and quantification of free OtV5 particles produced during the infection and lysis of *O. tauri*. Production of free viruses during infection experiments is often quantified with flow cytometry (e.g., Marie et al., 1999; Derelle et al., 2008). However, in our comparison of counts between VirusFISH and FCM, we observed that in late stages of infection of our non-axenic culture, an abundant population of mid-level fluorescence viruses, presumably phages, appeared in the cytogram just below the high-level fluorescence viruses, presumably OtV5 (Figure 5), but without a clear differentiation of the two close populations. OtV5 counts estimated by VirusFISH on the 0.02 μm anodisc filters and putative OtV5 particles estimated by FCM were in the same order of magnitude but slightly higher by FCM (7.4 × 10^8^ vs. 4.5 × 10^8^ virus ml^−1^, Figure 5) perhaps due to a certain overlap with the population of mid-level fluorescence phages. For this reason, we decided to use VirusFISH to quantify the production of OtV5 and also demonstrate the validity of this technique to visualize a desired virus within complex viral communities.

**Figure 6:**
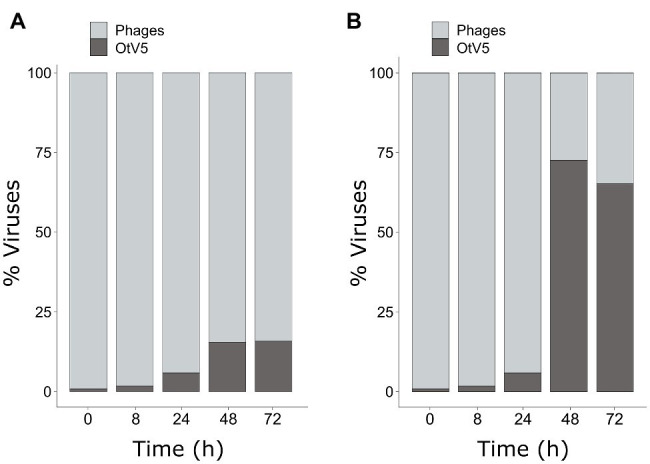
Dynamics of the proportion of OtV5 viruses in relation to total viruses (i.e., OtV5 and phages). **(A)** Counts on 0.02 μm filters. **(B)** Sum of the counts on the 0.02 μm filters and the viruses retained in the cellular matrix around the cells on the 0.2 μm filters. See Materials and Methods for details.

As mentioned above, SYBR Gold was used to counterstain the VirusFISH, which helped both to discriminate true OtV5 viruses from unspecific OtV5 probe signals (see Supplementary Figure S2) and from other phages (Figure 5B; Supplementary Figure S6). Only the VirusFISH red signal that overlapped with a SYBR Gold green fluorescence signal (yellowish particles in Figure 5B) was considered a true OtV5 particle. The number of free OtV5 viruses collected on the 0.02 μm filter when the major lysis occurred (48 h post-infection) was much lower than expected, representing only around 20% of total viruses (i.e., OtV5 and phages, Figure 6A). We realized that, after cell lysis, the organic matrix released from the cells trapped most OtV5 particles on the 0.2 μm filters (Supplementary Figure S3). We, therefore, summed together the free OtV5 particles detected on the 0.02 μm filters and the OtV5 estimated within the viral clouds around the cells (see Materials and Methods section for details) and obtained that the OtV5 particles produced represented around 75% of total viruses after the major cell lysis occurred (Figure 6B). Thus, we used this approach to estimate the OtV5 produced at 48 and 72 h post-infection. Since there was no visible cell lysis from 0 to 24 h, at those time points we only considered the free OtV5 viruses detected on the 0.02 μm filters.

**Figure 7:**
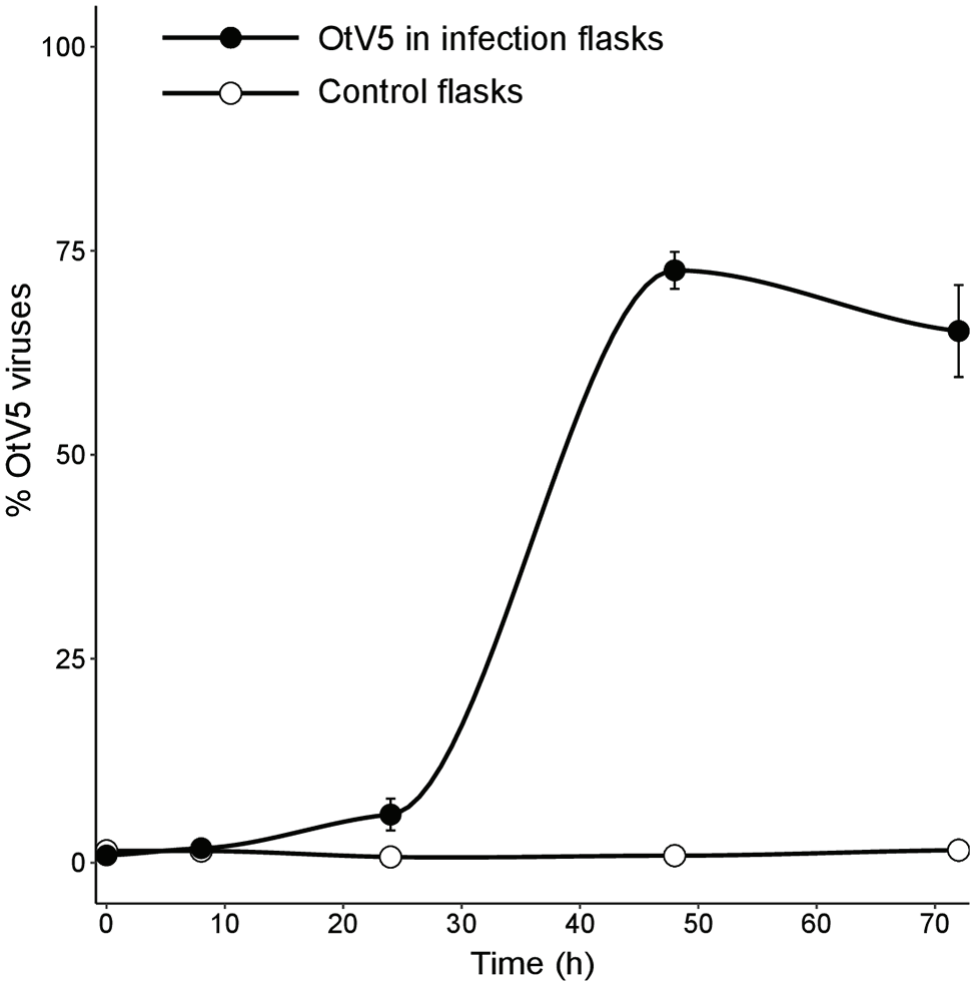
Dynamics of free viruses produced during the infection expressed as percentage of OtV5 with respect the total viral abundance (± standard error of the three replicates). Counts were done by epifluorescence microscopy considering the overlay of OtV5 and SYBR Gold signals and the retention of viruses on the 0.2 µm filters at 48 and 72 h (see Materials and Methods and Figure 6 for details).

Our results showed that before 24 h, some cell lysis had already occurred, as indicated by the slight, but detectable increase in OtV5 free particles at 24 h (Figures 6, 7). This agrees with the detection of 16% infected *O. tauri* cells at 24 h, which implies that at the MOI used some viral production had already taken place, as explained above. A drastic increase in viral abundance was observed after 24 h (Figure 7; Supplementary Figure S7), corresponding with the time the majority of cells was lysed. At 48 h, the number of free viruses reached a plateau, likely because most viral production had already occurred. Very low numbers of OtV5 viruses were detected in the control flasks (Figure 7; Supplementary Figure S7), likely false positives due to the long exposure time needed to acquire the images for the viral detection (Supplementary Figure S2). Yet, these false OtV5 counts were constant over the infection cycle, representing on average 0.5% (±0.1%) of total viruses (Figure 7). The fraction of OtV5 within the total viral community in the infection flasks ranged from 0.9% (±0.2%) at 0 h when viruses were inoculated to 72.1% (±5.6%) at 48 h when almost all *O. tauri* cells were lysed (Figures 3, 7).

## Discussion

Several studies have dealt with the virus-host relationships of the four clades of *Ostreococcus* spp. (*O. tauri*, *O. lucimarinus*, *O. mediterraneus*, and clade B; Guillou et al., 2004), and our knowledge on these systems is continuously expanding (Weynberg et al., 2017). From these studies, only a few focused on the infection dynamics (e.g., Derelle et al., 2008; Heath and Collins, 2016). Most of the work has been directed toward understanding the virus-host interaction at the molecular level (e.g., Derelle et al., 2008; Weynberg et al., 2011; Clerissi et al., 2012), unveiling, for instance, interesting information on the host resistance mechanisms to viruses (Thomas, 2011; Heath and Collins, 2016; Yau et al., 2016). However, to understand the impact of viruses on the ecology of *Ostreococcus* spp., it is crucial to develop techniques that enable monitoring the host-virus interactions at the single cell level, with the ultimate goal to apply them in complex natural communities. We designed probes to detect OtV5, but the alignment of the probes with other Prasinovirus genomes showed that they can potentially label all 11 genome-sequenced *Ostreococcus* spp. viruses (Supplementary Tables S2, S3), except OtV6, which is evolutionarily distinct (Monier et al., 2017). Thus, our technique may help fostering our knowledge on the role of viruses in the control of the abundance of the cosmopolitan *Ostreococcus* spp.

Unlike flow cytometry measurements and plaque-forming unit assays, which only give absolute cell and virus counts, VirusFISH allowed to follow the whole process of infection and shed light on what was happening previous to culture clearance, unveiling that infection was faster than what it could be inferred from only cell or free virus counts. It showed that, despite the adsorption efficiency 8 h after virus inoculation was low, with only 0.2% of infected cells, this value increased to 16% at 24 h, and a fast lysis of the culture occurred before 48 h (Figures 2B, 3).

Another valuable application of VirusFISH was to determine the free viral particles released during infection, discriminating the true OtV5 from phages and other unspecific particles and improving the estimation of viral production in non-axenic cultures. The latter is commonly done through flow cytometry (e.g., Derelle et al., 2008; Brown and Bidle, 2014), but may have some problems due to the presence of mid-level fluorescence phages at the late stages of infection that appear with a certain overlap in the cytogram with the putative OtV5 population (Figure 5), potentially resulting in an overestimation of OtV5 viral production. We thus showed the utility of VirusFISH to detect our virus of interest within a complex viral community.

VirusFISH also unveiled that a large proportion of the viruses produced get trapped within the organic matrix released during cell lysis, which affected the quantification of the free viruses produced. It is important to note that retention of OtV5 on the 0.2 μm filters will affect the same way OtV5 counts regardless of the technique used to count them (flow cytometry, plaque assay, or VirusFISH), as filtration through 0.2 μm is the common procedure to remove cells and cell debris in all three techniques prior to viral counting. Thus, visualization with VirusFISH revealed a process that affects estimations of viral production and burst size values and that should be considered for future studies on viral infection dynamics.

Given the relatively low amount of OtV5 particles detected with VirusFISH, it could be argued that VirusFISH may only be counting non-encapsidated viruses. However, we find this highly unlikely. VirusFISH is applied to viral particles retained in a 0.02 μm filter, whereas naked DNA would pass through the filter. Furthermore, we obtained similar counts of free OtV5 particles with flow cytometry and VirusFISH (Figure 5), and encapsidated viruses were seen surrounding cell debris in electron microscopy images (Figure 1), whereas no intermediate forms such as immature viruses were observed.

Although it was not the goal of our study due to the tiny size of *Ostreococcus*, VirusFISH could be potentially used for visualizing the dynamics of the viruses within the eclipse phase of infection, something that is not feasible with other methods like Transmission Electron Microscopy.

### Methodological Aspects to Be Considered for Phototrophic Eukaryotes and Our Particular *O. tauri* System

One of the best fluorochromes to label gene probes is Alexa594 (Barrero-Canosa et al., 2017), which emits red fluorescence when excited with orange light. However, the chloroplasts of photosynthetic microbes also emit red fluorescence under the same light, hampering the detection of viral signals. We solved this technical issue by removing the cellular pigments with a combination of alcohol treatments, as described in the Materials and Methods section.

The filter pore size also needs to be considered during VirusFISH experiments. *Ostreococcus* cells have a typical diameter of 1 μm, but we observed that some cells passed through 0.6 μm filters, most likely because their cellular membranes are very flexible. This resulted in the loss of more than half of the cells during filtration. Consequently, we recommend the use of filters with a pore size of 0.4 or 0.2 μm when working with picoeukaryotes.

### Modifications of VirusFISH With Respect to the Published Protocols of PhageFISH and Direct-geneFISH

VirusFISH represents a combination between phageFISH and direct-geneFISH. It uses CARD-FISH to identify the unicellular eukaryotic host, similar to phageFISH, and uses a mixture of polynucleotide probes directly labeled with a fluorochrome to target viral genes, similar to the direct-geneFISH protocol. CARD-FISH was used because its signal amplification step enables the detection of cells with low ribosome content. *O. tauri* and all Mamiellophyceae have a small cytoplasm due to the relatively big size of the organelles (Henderson et al., 2007), and therefore, their ribosomal abundance is low, and CARD-FISH enhances the cellular visualization. We also incorporated a step of embedding the filters in agarose to avoid cell losses in downstream manipulations of the filter portions. Furthermore, because *O. tauri* lacks a cell wall, the permeabilization step usually needed in the CARD-FISH protocols (Pernthaler et al., 2002) was omitted. On the other hand, a treatment to completely remove cell pigments was required, as mentioned above. Finally, compared to the direct-geneFISH protocol, we reduced the Alexa594 fluorochrome volume to label the viral gene probes in order to reduce economical costs but obtaining equally optimal results (see details in the Materials and Methods section).

### VirusFISH vs. Other Approaches to Follow Virus-Host Dynamics

Currently available methods to assess the dynamics between host and viruses during infection are (i) the frequency of visibly infected cells (FVIC; Wommack and Colwell, 2000), (ii) quantitative PCR (qPCR) of viral genes (Chen and Suttle, 1995; Larsen et al., 2008; Matteson et al., 2011), and (iii) the plaque assay, for counting plaque forming units (PFU; Brussaard et al., 2016). Compared with these methods, VirusFISH brings further advantages. For example, FVIC reports the fraction of infected host cells, but only detects those cells in the late stage of infection. PFU requires that the host is available in culture, and although both PFU and qPCR allow following the infection dynamics, they lack the ability to quantify the fraction of infected cells. In comparison, VirusFISH allows (i) the identification of both host and virus, using 18S rRNA and specific probes that target viral genes, features particularly advantageous in non-axenic cultures of unicellular eukaryotes or in environmental samples; (ii) the quantification of the total and relative abundance of the host cells; (iii) quantification of the total and relative abundance of virus-infected cells, independent of the stage of infection; and (iv) quantification of released viral particles. Furthermore, VirusFISH could potentially be used to discriminate the different stages of infection, as it has been done with phageFISH (Allers et al., 2013).

Some other approaches have arisen in the last decade to unveil virus-host interactions, like the polony method (Baran et al., 2018) or the microfluidic digital PCR (Tadmor et al., 2011). The novel polony method is a culture independent technique based on a single molecule PCR. Using degenerate primers, it allows the determination of the abundance of a given viral group and its degree of diversity, discriminating between different viral families or genera and their host. This high-throughput approach has enabled the quantitative assessment of thousands of viruses in a single sample from both aquatic and terrestrial environments (Baran et al., 2018). Thus, the polony method is a powerful approach to detect virus-host interactions in a cost-effective and relatively simple manner, but similar to VirusFISH, it requires the knowledge of the hosts and the genome of the viral target to design the probes. However, although the VirusFISH approach is not as high-throughput as the polony method, it has the advantage that it allows monitoring viral infection dynamics, so we can see when the infection is taking place, how many cells are infected at different times, and how the infection progresses. Likewise, it allows visualizing early phases of infection or latent infections that do not result in a massive lysis of the culture.

Moreover, since VirusFISH uses microscopy observations, it enables the study of the heterogeneity of the infection within the host population, with the potential to extend its use to assessing specific virus-host interactions in complex natural communities.

### Free Viral Abundances

The abundance of free viruses has been traditionally assessed through (i) TEM of uranyl acetate stained virus particles (Malenovska, 2013) and (ii) by epifluorescence microscopy (Hennes et al., 1995) or flow cytometry (Marie et al., 1999) of SYBR Green stained viruses. On the other hand, infective viruses are traditionally quantified by plaque assays (Suttle and Chen, 1992). Each of the above methods have limitations: (i) TEM is a time consuming, difficult to perform quantitatively, and expensive technique, (ii) SYBR staining followed by epifluorescence microscopy or flow cytometry does not distinguish between infective and non-infective viruses, and it is impossible to identify the virus of interest within a complex viral community, and (iii) the plaque-assay is constrained to cultivable hosts and their viruses. With VirusFISH we achieved the detection of specific free viruses in a relatively fast way, with no requirements of specialized equipment, or extremely expensive reagents. Allers et al. (2013) also applied phageFISH to visualize free viral particles, but they immobilized the viral lysate on glass slides, which can potentially lead to virus losses during the hybridization process. We tried to overcome this issue by collecting and counting the free viruses on 0.02 μm anodisc filters decreasing the risk of viral losses during the hybridization process due to a better retention. Additionally, one filter can be used several times (by cutting the filter in portions) and kept at −80°C for years. We also showed that discriminating our virus of interest from phages in non-axenic cultures is crucial for assessing viral production over time.

## Conclusion

In summary, in this study, we developed VirusFISH to detect virus-host interaction in *O. tauri*. This technique allowed us to visualize and follow the dynamics of the OtV5 viral infection of *O. tauri* until the complete lysis of the culture. Also, VirusFISH enabled the calculation of the viral production during infection, discriminating OtV5 viruses from the phages present in the non-axenic culture. Our designed probes could potentially target most *Ostreococcus* viruses, except for OtV6, representing a valuable tool to address virus-host interactions in these cosmopolitan marine picoeukaryotes. We strongly believe that VirusFISH presents great prospects to address infection dynamics in nature, and it will foster our understanding on the impact of viruses in eukaryotic populations. Furthermore, this technique can be easily adapted and implemented on any other model system.

## Data Availability Statement

All datasets generated for this study are included in the article/Supplementary Material.

## Author Contributions

YC: data curation, formal analysis, investigation, methodology, visualization, writing original draft, and review and editing. MS: data curation, formal analysis, investigation, methodology, supervision, validation, and writing – review and editing. IF: formal analysis, investigation, methodology, visualization, and writing – review and editing. NG: writing – review and editing. SY: data curation, formal analysis, and writing – review and editing. CM: methodology and writing – review and editing. DV: data curation, formal analysis, investigation, methodology, project administration, resources, supervision, validation, and writing – review and editing. All authors contributed to the article and approved the submitted version.

### Conflict of Interest

The authors declare that the research was conducted in the absence of any commercial or financial relationships that could be construed as a potential conflict of interest.

## Acknowledgments

We thank the Unitat de Criomicroscopia Electrònica (Centres Científics i Tecnològics, Universitat de Barcelona) for the electronic transmission images. This work is included as a chapter in the PhD thesis of YC (Castillo, 2019).

## Supplementary Material

The Supplementary Material for this article can be found online at: https://www.frontiersin.org/articles/10.3389/fmicb.2020.01559/full#supplementary-material.
