# Supplementary material for: Visualization of Viral Infection Dynamics in a Unicellular Eukaryote and Quantification of Viral Production Using Virus Fluorescence in situ Hybridization
Source: Front Microbiol. 2020 Jul 17;11:1559. doi: 10.3389/fmicb.2020.01559 (PMC7379908; doi:10.3389/fmicb.2020.01559)
Supplement: Supplementary file 1 [file Data_Sheet_1.PDF]

Supplemental Information for:

Visualization of viral infection dynamics in a unicellular eukaryote and quantification of viral production using VirusFISH

Yaiza M. Castillo[1], Marta Sebastián[1,2], Irene Forn[1], Nigel Grimsley[3], Sheree Yau[1,3], Cristina Moraru[4] and Dolors Vaqué[1]

Author affiliations:

[1]Department of Marine Biology and Oceanography, Institute of Marine Sciences (CSIC), Barcelona, Catalonia, Spain.
[2]Institute of Oceanography and Global Change (IOCAG), University of Las Palmas de Gran Canaria (ULPGC), Las Palmas de Gran Canaria, Spain.
[3]Integrative Biology of Marine Organisms (BIOM), Sorbonne University, CNRS, Oceanographic Observatory of Banyuls, Banyuls-sur-Mer, France.
[4]Department of Biology of Geological Processes, Institute for Chemistry and Biology of the Marine Environment, Oldenbug, Germany

Description of Supporting information content:
Supporting information is gathered in a single file which contains (in the following order):
- 3 supplementary tables
- 7 supplementary figures
- A step-by-step VirusFISH protocol

Table of Contents:

Table S1	Page 2
Table S2	Page 3
Table S3	Page 4
Figure S1	Page 5
Figure S2	Page 6
Figure S3	Page 7
Figure S4	Page 8
Figure S5	Page 9
Figure S6	Page 10
Figure S7	Page 11
Step-by-step protocol	Page 12

Table S1. Position of the 11 probes in the OtV5 genome and the nucleotide sequence of the primers used to synthetize the viral probes.

Probe	Probe position in genome (bp) (Start / End)	Forward Primer sequence	Reverse Primer sequence
#1	102,846 / 103,145	CGAAGACGACAGCGAAGACCA	TCAGCCCAGGCGTTCTCTTGA
#2	103,148 / 103,447	GCCAAAAAAAGGGCGGCTGGGA	GCGAACTCATGACGGACTGTAC
#3	103,459 / 103,758	GCATCGTGGATGAGTTGCTCAA	TTGGTGGCGATGAGGCCAA
#4	103,765 / 104,064	TAAGGAGCCCCTCCCTGAT	GCGTTCTTTGTGCTAATTTCGC
#5	104,179 /104,478	GGGAAGCCAATTTGTTGGCGTG	AAACCTCGCGATGTTGGCTGCG
#6	104,479 /104,778	TGGGCACGACCCTTCTTCATCA	CTCAAGTGTCGCCGATGGTCAA
#7	104,792 / 105,091	CGATCACTTTTTCGGAGAGTTG	CATGATCCCCTTTGTTGGTTTA
#8	105,174 / 105,473	TATTTGGAATACCCTTACCCGT	TAAAGAGTGTTCGTTTCGTCAC
#9	105,631 / 105,930	GAGGAGGAAACGCTTTCCAG	CGCTACCTCCTGATCAAGAAGA
#10	105,941 / 106,240	TATGGCCCCTTCTCCGAGAAA	CGCGGAACTTTCTGAATTCCTC
#11	106,545 / 106,844	AGTTCTGCGGGGTGCGCAAA	ACGAGCTTGGTGAGGGCCTTA

Table S2. Alignment of our designed viral probes against Prasinovirus genomes and the outgroup PbCV1 (Chlorovirus) using BLAST. Shaded cells indicate identity higher than 80%, and coverage larger than 90%. Given than the probes are 300 bp long and all of them are combined for the hybridization, they may serve to visualize the virus-host interactions of all the *Ostreococcus* virus described in the table, except for OtV6. Accession numbers are shown in Table S3.

	**Probe 1**		**Probe 2**		**Probe 3**		**Probe 4**		**Probe 5**		**Probe 6**		**Probe 7**		**Probe 8**		**Probe 9**		**Probe 10**		**Probe 11**	
	**Id (%)**	**Cov (%)**	**Id (%)**	**Cov (%)**	**Id (%)**	**Cov (%)**	**Id (%)**	**Cov (%)**	**Id (%)**	**Cov (%)**	**Id (%)**	**Cov (%)**	**Id (%)**	**Cov (%)**	**Id (%)**	**Cov (%)**	**Id (%)**	**Cov (%)**	**Id (%)**	**Cov (%)**	**Id (%)**	**Cov (%)**
**OtV5**	100	100	100	100	100	100	100	100	100	100	100	100	100	100	100	100	100	100	100	100	100	100
**OtV1**	97.7	100	98.7	100	98.7	100	99	100	98.3	100	99.3	100	99.7	100	98.3	100	98.7	100	98.7	100	96.7	100
**OtV2**	85.3	100	80.8	98	82	100	89.3	99	88.8	100	89.6	99	85.5	99	82.2	93	84.6	99	84.6	99	82	100
**OtV6**	77.4	100	0	0	74.8	90	83.2	99	78.6	93	82.2	99	75.3	97	0	0	0	0	0	0	84.1	35
**OlV1**	86.7	100	80.7	98	81.8	100	85.6	99	84	100	83	99	83	98	82	98	85.6	99	85.2	99	76.3	99
**OlV2**	85.3	100	84.2	98	82.3	100	91	99	89.1	100	89.6	99	85.5	99	83.6	93	99	84.6	84	99	80.7	100
**OlV3**	85.3	100	84.5	98	82	100	91.3	99	91.1	100	89	99	85.9	99	83.3	93	84	99	85	99	81	100
**OlV4**	0	0	79.7	87	81.8	100	85.6	99	85.3	100	82	99	82.7	98	82.3	98	85.6	99	86	99	74.4	100
**OlV5**	85	100	80.7	98	82.7	100	89.9	99	89.1	100	89.3	99	85.2	99	81.8	97	85	99	84.6	99	83	100
**OlV6**	85.3	100	80.6	97	82.7	100	89.9	99	89.4	100	89.3	99	85.2	99	81.8	93	85	99	84.6	99	82	100
**OlV7**	85.3	100	80.1	98	81.8	199	86	99	85.3	100	83.7	99	82.2	99	83.6	93	85	99	85.3	99	74.6	99
**OmV1**	95.7	100	98.3	100	98.3	99	99.7	99	97.7	100	99.7	100	99	100	99.3	100	99.3	100	99.3	100	98.3	100
**OmV2**	84	100	78	92	86.7	87	88.6	99	87.8	99	89	99	88.3	99	80.7	91	87	99	85.6	99	87.8	98
**MpV1**	74.8	99	81.6	25	69.3	90	73.7	76	71.8	93	76	82	81.5	59	0	0	86.5	35	0	0	76	25
**BpV1**	71.1	74	86.7	20	66.3	33	0	0	79.7	42	0	0	0	0	0	0	73.1	22	0	0	0	0
**PbCV**	0	0	0	0	0	0	0	0	0	0	0	0	0	0	0	0	0	0	0	0	0	0

Abbreviations: Id, Identity; Cov, Coverage; OtV, *Ostreococcus tauri* virus; OlV, *Ostreococcus lucimarinus* virus; OmV, *Ostreococcus mediterraneus* virus; MpV, *Micromonas pusilla* virus; BpV, *Bathycoccus prasinos* virus; PbCV, *Paramecium bursaria Chlorella* virus 1.

Table S3. Genbank accession of Prasinovirus genomes and the outgroup PbCV1 (Chlorovirus) used in Table S2.

Virus name	Accession number
OtV5	EU304328.2
OtV1	FN386611.1
OtV2	FN600414.1
OtV6	JN225873.1
OlV1	MK514405.1
OlV2	KP874736.1
OlV3	HQ633060.1
OlV4	JF974316.1
OlV5	HQ632827.1
OlV6	HQ633059.1
OlV7	MK514406.1
OmV1	KP874735.1
OmV2	MN688676.1
MpV1	HM004429.1
BpV1	HM004432.1
PbCV	NC_000852.1

Abbreviations: OtV, *Ostreococcus tauri* virus; OlV, *Ostreococcus lucimarinus* virus; OmV, *Ostreococcus mediterraneus* virus; MpV, *Micromonas pusilla* virus; BpV, *Bathycoccus prasinos* virus; PbCV, *Paramecium bursaria* Chlorella virus 1.

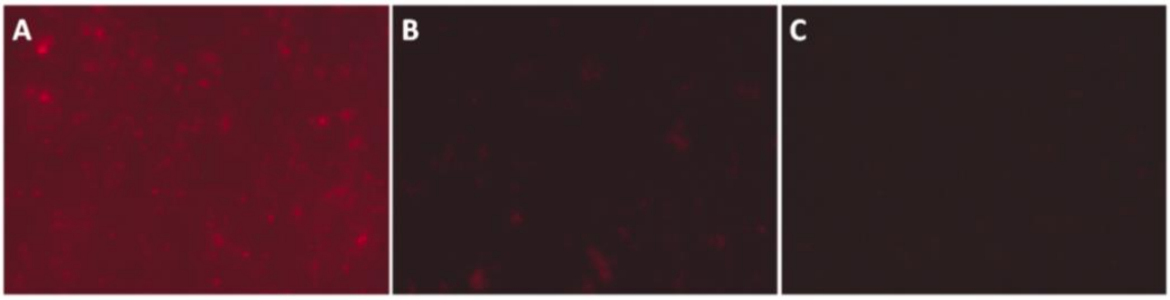

Figure S1. Tests of *Ostreococcus tauri* RCC4221 culture for chlorophyll pigments removal. **A.** Negative control, no alcohol treatment. Chlorophyll is clearly visible. **B.** Cell culture treated for 1 hour with ethanol 97%. Chlorophyll is visually reduced, but not completely eliminated. **C.** Cell culture treated for 1 hour with ethanol 96% followed by 1 hour pure methanol. Chlorophyll is completely removed and almost no red background is visible.

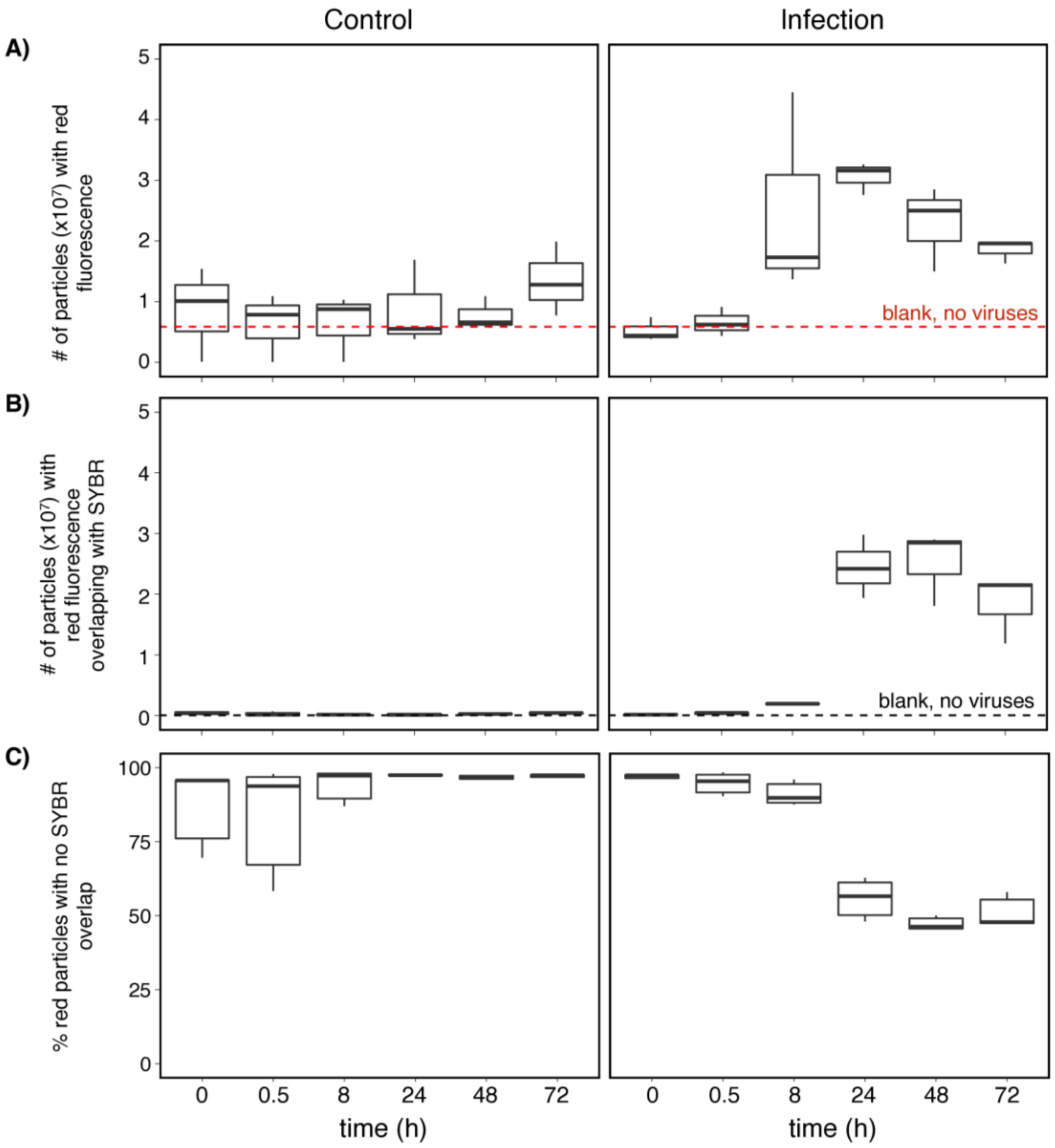

Figure S2. Analyses of non-specific red fluorescence in the 0.02 µm pore size anodisc filters. **a)** Red fluorescent particles in the control and the infection flasks, the dotted line represents the number of particles detected in a blank filter (no sample) that underwent the same hybridization procedure as the samples. **b)** Red fluorescent particles that co-localized with a SYBR gold particle (what we considered true viruses). **c)** proportion of red particles that co-localized with a SYBR gold particle. **Note that false positives decreased in the infection flasks as the infection progressed**. These virus counts only included the OtV5 collected onto the 0.02 µm filters, but a large number of OtV5 particles got retained in the 0.2 µm filters (see main text). This is the reason why the proportion of true viruses in the lower panel is only 50%.

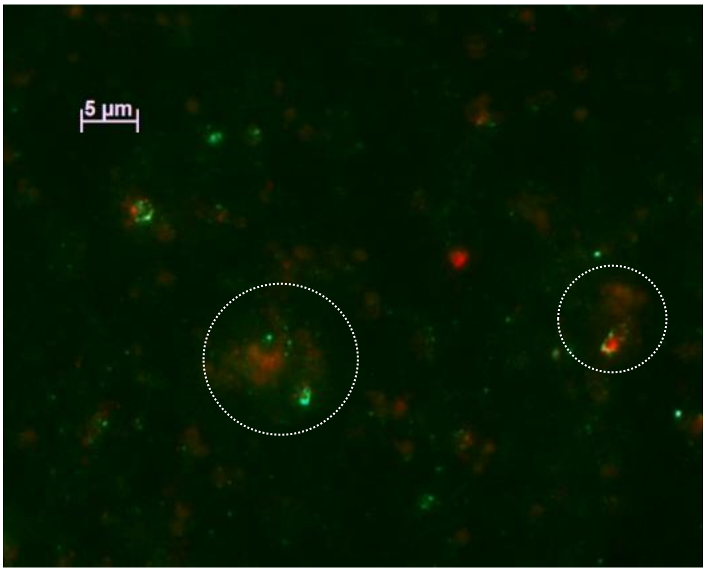

Figure S3. Micrograph showing viral clouds around lysed cells (within the dotted circles) at time 48 h post-infection.

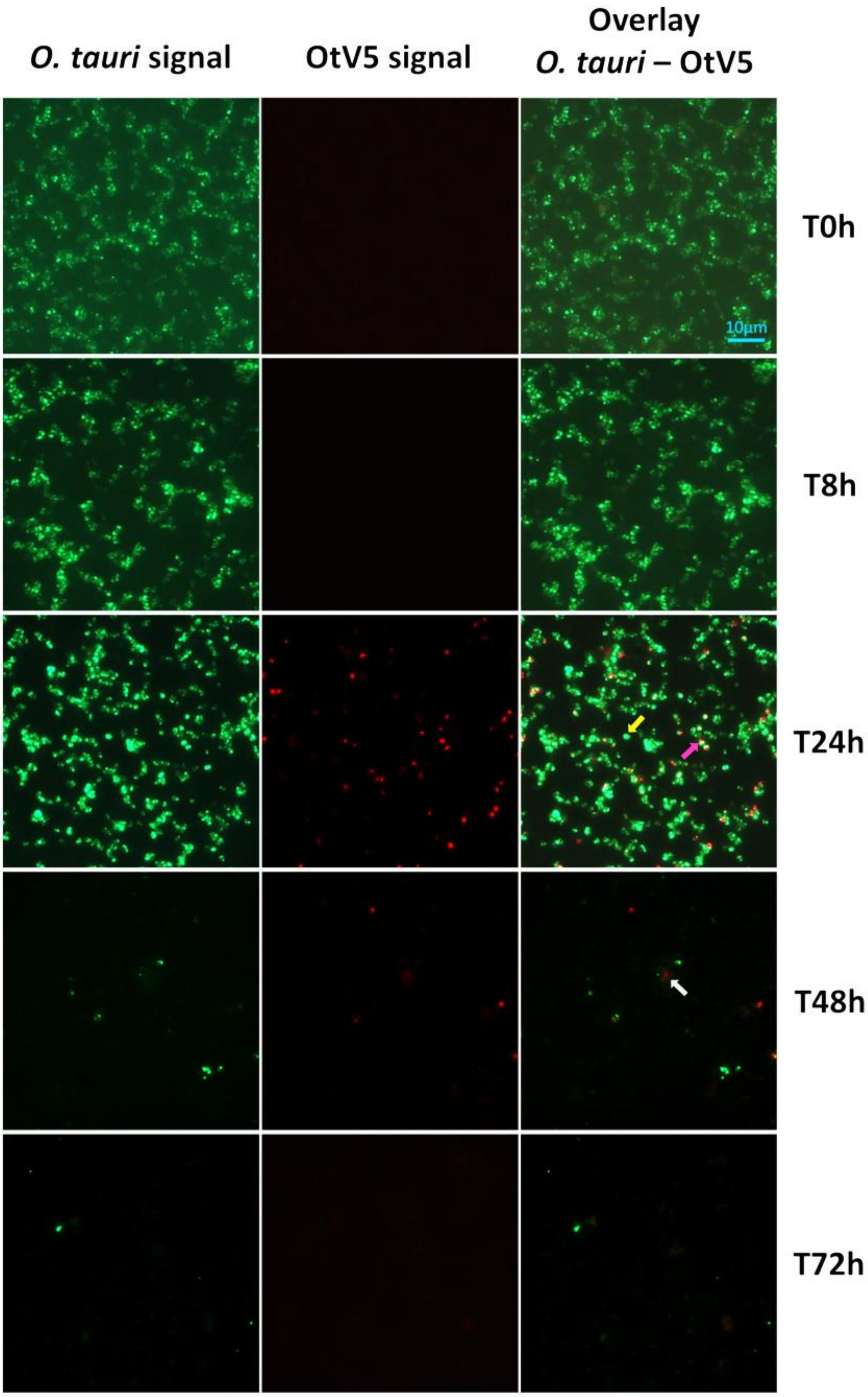

Figure S4. Micrographs of the evolution of the infection from time 0h to 72h. Left: *O. tauri* only. Centre: OtV5 only. Right column: overlay of *O. tauri* host cells in green (Alexa488) and virus in red (Alexa594). Yellow arrow: non-infected *O. tauri*; pink arrow: infected *O. tauri*; grey arrow: cloud of viruses retained by the organic matter released during cell lysis.

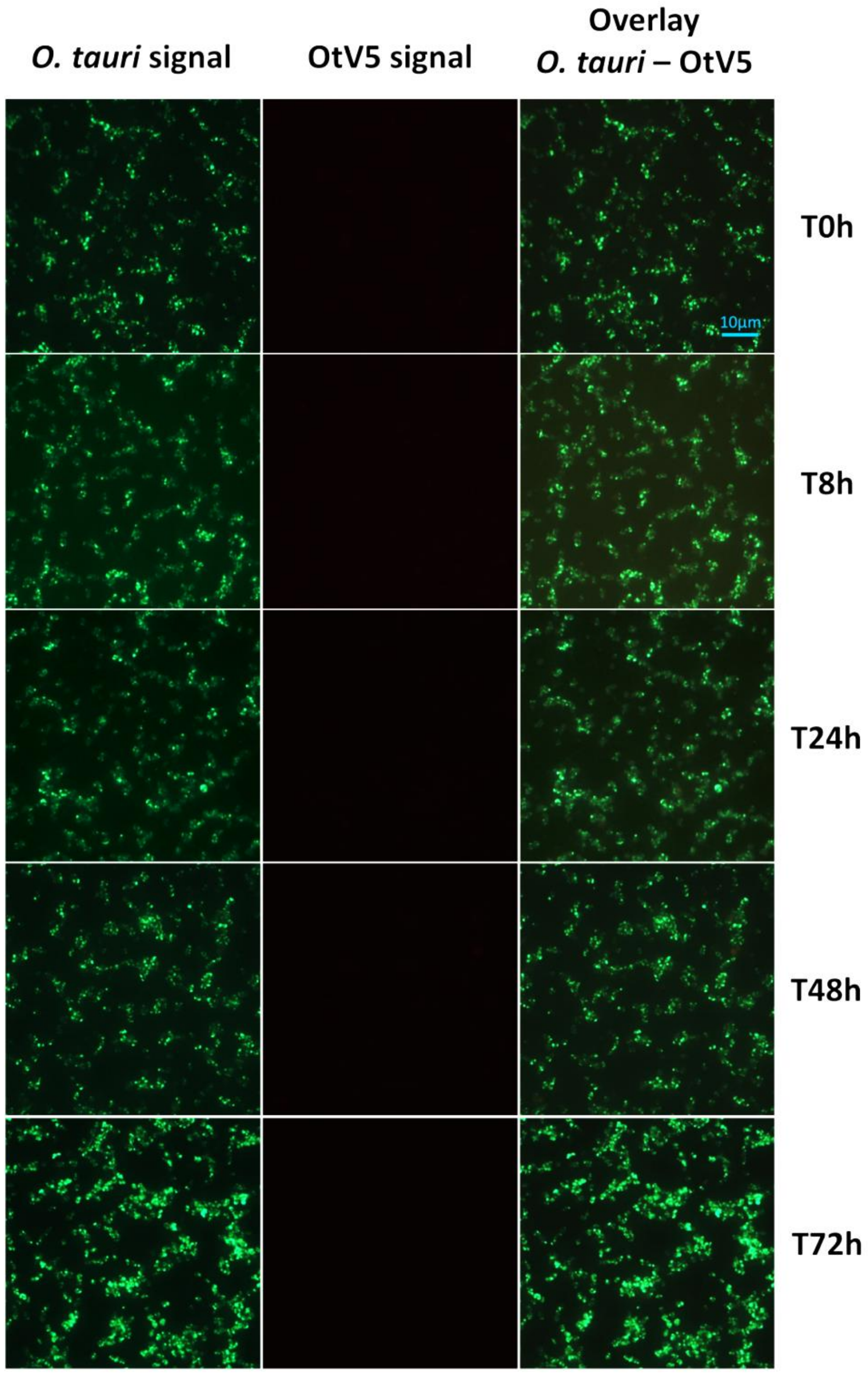

Figure S5. Micrographs of the evolution of the control flasks from time 0h to 72h. Left: CARD-FISH against *O. tauri* (Alexa488). Centre: VirusFISH against OtV5 viruses (Alexa594). Note there is no viral probes hybridation nor false positives. Right column: overlay of both hybridization colors (Alexa488 and Alexa594).

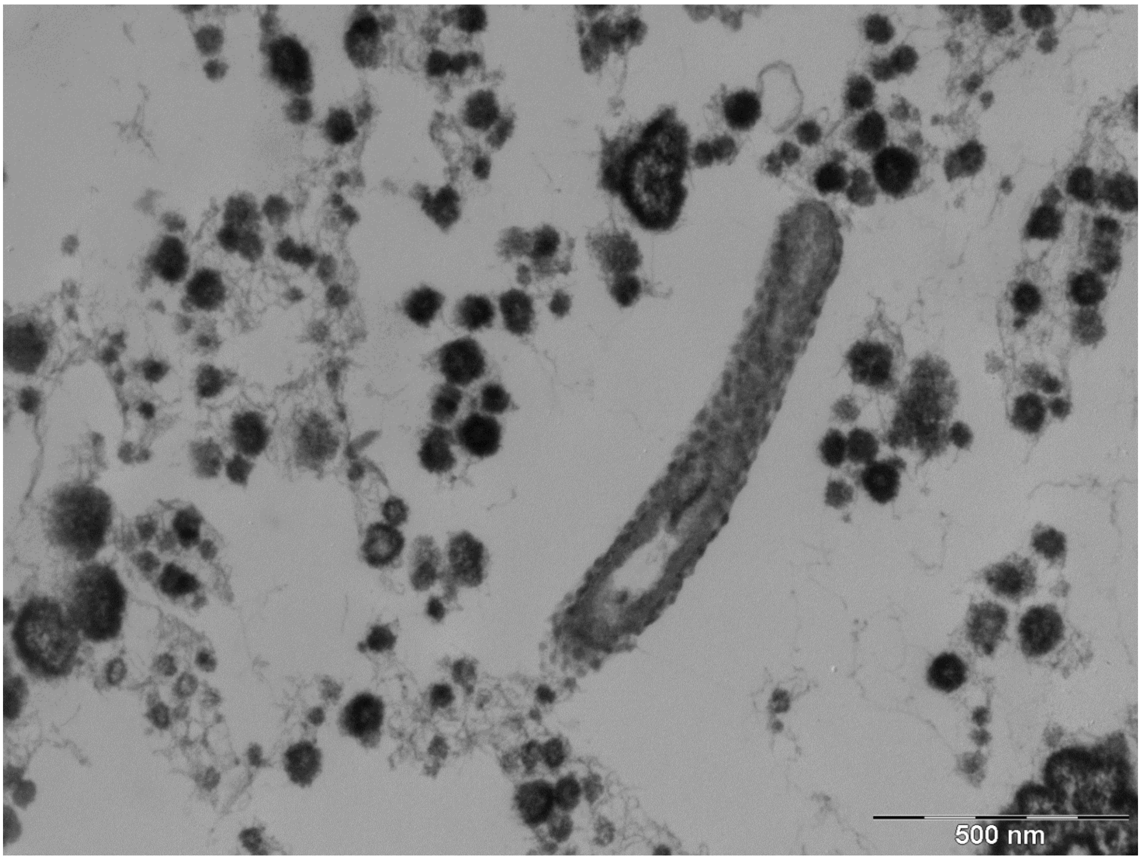

Figure S6. Transmission electron microscopy image of an infected bacteria releasing phages in the non-axenic *O. tauri* culture.

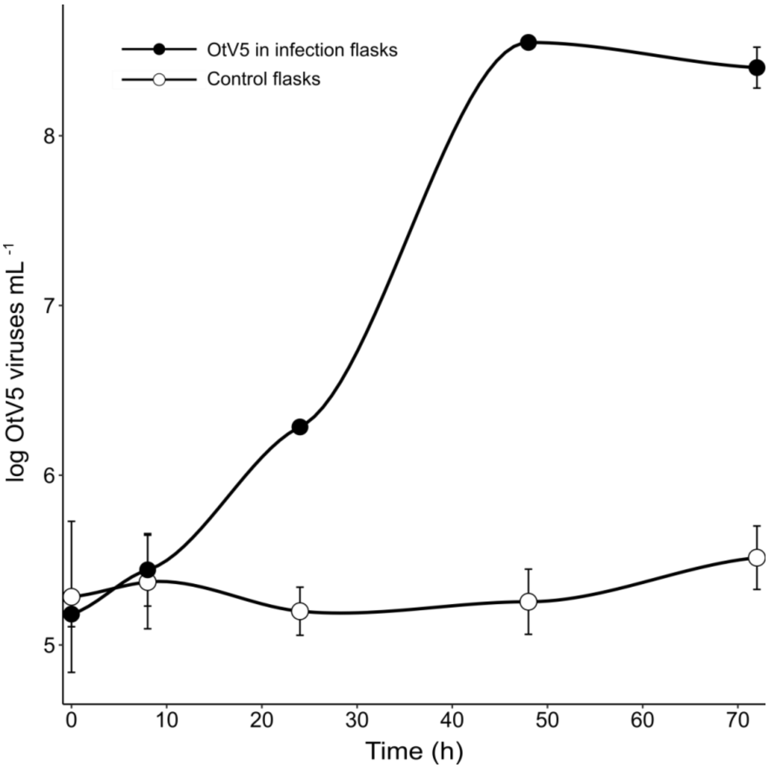

Figure S7. Dynamics of OtV5 particles abundance (average±SE) determined by VirusFISH. Black dots represent OtV5 particles in infection flasks. White dots represent the basal false positive OtV5 viruses (mean of $3 \cdot 10^5$ virus ml^{-1}). See Figure S2 for more information on these false positives, which represented only 0.5% of total viruses.

CARD-FISH/VirusFISH PROTOCOL

1. Virus probes design and synthesis

The VirusFISH method requires that we design, synthetize and label the probes that will hybridize to our virus of interest. In this section we explain what is needed for this purpose.

1.1. Primers design

The VirusFISH probes will consist on ~10 probes (300pb each) that will be synthetized by PCR. For this, we need to design, together with the probes, the primers that will be used in the PCR. Ad hoc, we use the genePROBER program that can be found in the following link: https://gene-prober.icbm.de

STEP1:

- Input two fasta files: one with the targeted virus and one both with the target and non-target sequences
- In the field "Name of contig with target in ALL sequences file" write the name of the virus in the fasta file without the fasta ">" symbol.
- In the field "Target start" write "1".
- In the field "Target end" write the viral genome size in bp (e.g.: Otv5 → 186,713bp)

STEP 2:

- Search for similarity between the virus and the host to avoid selecting similar regions that will give false positives.

STEP3

- Select a range of the viral genome that does not match a region similar to the host genome to avoid unspecific binding of the probes (see STEP2).

STEP4:

- Progressively remove unsuited polynucleotides based on polynucleotide and primer characteristics

Note: if not specified keep the default values.

- 4a) select polynucleotide GC range (recommended 5% range)
- 4b) select primer parameters (GC%, Tm range, dimers)
- 4c) select polynucleotides by removing those which potentially bind to non-specific targets
- 4d) Select polynucleotides by removing those whose primers are unspecific or inefficient.

Choose those primers pairs that allow to have probes with distances below 100bp to the next probe (exceptionally it can be 900bp between probes). The best primers will be those closer to 50% of GC and similar Tm.

1.2. Primers pre-treatment

Primers are sold lyophilized. Hydrate them with sterile MQ water to a final concentration of 100 µM. Make aliquots and store the original stock. Keep both at -20 ºC.

1.3. Selection of the annealing temperature of the primers

Prepare a master mix (MM) according to the number of primers and annealing temperatures to test, considering a 10 µl PCR reaction. Do the PCR for a minimum of 10 primer sets to obtain 10 virus probes.
Volume's template of MM (for a 10 µl-reaction final volume):

	Total volume (µl)	Final concentration
10x reaction buffer	1	
Mg 50mM	0.3	1.5mM
dNTP 10mM	0.2	200 µM
Taq 5U/µl	0.1	0.05U/µl
DNA Template	2pg	
MQ	to 10 µl	

Template: 2pg of viral genomic DNA for each 10 µl of reaction. Calculate the amount of DNA to add to your master mix.
Distribute the master mix in your PCR tubes.
Add the primers to each 10 µl-reaction to yield 1 µM.

PCR settings:

<table>
<tr><td></td><td>95ºC</td><td>5min</td><td></td></tr>
<tr><td>Denaturing</td><td>95ºC</td><td>1min</td><td rowspan="3">x30</td></tr>
<tr><td>Annealing</td><td>X temperature</td><td>30s</td></tr>
<tr><td>Elongation</td><td>72ºC</td><td>30s</td></tr>
<tr><td></td><td>72ºC</td><td>10min</td><td></td></tr>
<tr><td></td><td>4ºC</td><td>∞</td><td></td></tr>
</table>

Perform a gradient PCR to choose the optimal annealing temperature (usually ~5 ºC lower than the primers melting temperature). Choose the temperature that gives the higher quantity of PCR product with a unique band in the electrophoresis (excepting the small dimers).

Take into account only those primers with a similar annealing temperature and discard the rest.

1.4. Probes synthesis (PCR)

Do several PCR to achieve a total volume of ~400 µl for each probe (i.e. 8 reactions of 50 µl). If the product gives a dark band in the electrophoresis gel then you can reduce it to ~300 µl.

1.5. Probes purification

Purify the synthesized probes during the PCR using a purification column [QIAquick PCR Purification kit (50, Qiagen, Germany; cat. no.: 28104)]. Purify all PCR reactions for one probe in a single column (it helps to concentrate more the probe), but use a different column for each probe.

Follow the QIAquick PCR Purification kit protocol:

1. Add 5 volumes of union buffer (PB) to 1 volume of PCR reaction and mix (e.g.: 250 µl of PB for 50 µl of the PCR product).
    a. Place the mix in the QIAquick column.
2. Centrifuge 30-60 seconds at 13000rpm.
    a. Repeat steps 1 and 2 with all the PCR reactions. One tube for each of the probes.
3. Wash with 750 µl of PE buffer. Centrifuge 30-60s at 13000rpm.
4. Centrifuge again for 60s at 13000rpm with the same tube to remove the residual washing buffer.
5. Place the column in a new Eppendorf tube. Add 30-50 µl of Tris-EDTA buffer (5mM-1mM respectively, pH 8), do not use the elution buffer associated to the kit. Add the buffer in the middle of the membrane (do not touch the membrane).
    a. Centrifuge 1 minute at 13000 rpm.
    b. Keep the eluted product at -20 ºC.

Test the purification and concentration of the probe:

- Run an electrophoresis gel to check for the quality of the probes with 1 µl of purified PCR product.
- Test also the concentration using a NanoDrop 1000 (Fisher Thermo Scientific) with 1 µl of each purified PCR product. Each probe must have a concentration of ~100ng/µl or higher. The final probe mixture must have equal contribution of each of the probes and 1000ng (100ng/µl final concentration).
    - Note: as more probes are produced the concentration requirements are reduced, to calculate the minimum concentration required for each probe you can use this formula:

1000ng / # number of probes = minimum amount of each probe

- Mix all the probes in equal concentrations. To determine the volume of each probe to add to the mixture:

Minimum amount of each probe (ng) / concentration of the probe (ng/µl) = probe volume (µl)

- If the total volume, once all the probes are mixed, is below 10 µl, add the C component included in the Alexa kit (C component is equivalent to the buffer Tris-EDTA, 5mM-1mM, pH 8 that was used in the probes purification step) to reach the 10 µl volume desired.

- Note: if the probes are not very concentrated the final volume in the mixture will be over 10 µl. The maximum volume for the next step should not exceed 11 µl. To avoid this, synthetize again those probes that have a very low concentration.

From here on all the steps will be done using the probes mixture

1.6. Labeling process

Use the Ulysis Labeling Kit (Life technologies, USA; U21652, U21654, U1660).

Alexa594 is provided dehydrated: add 100 µl B Component (50% DMF o DMSO) and vortex. You can keep it at 4 ºC up to 6 months.

Labeling protocol (using the thermocycler):

1. Separate the two DNA strands:
    a. 95°C, 5min
    b. Spin.
2. Place the reaction fast on ice:
    a. 10min
    b. Spin.
3. Add the labeling kit (Alexa594):
    a. 15 µl of Alexa594
    b. Incubate for 80°C, 30min
    c. Spin.
4. Place it fast on ice to stop the reaction.

1.7. Probes mixture purification (cleaning of excess of Alexa594)

Use the Micro Bio-spin chromatography columns P-30 (Bio-Rad, California, USA, cat.no. 732-6202) to purify the probes mixture.

Protocol:

1. Invert the column sharply several times to resuspend the settled gel. Tap de column to remove all air bubbles. Snap off the tip and place the column in a 2.0 ml microcentrifuge tube (supplied with the kit). Remove the cap. If the buffer does not begin to flow from the column, push de cap back on the column and remove it again to start the flow. Allow the excess packing buffer to drain by gravity to the top of the gel bed (about 2 min). Discard the drained buffer and place the column back into the 2.0 ml tube.
2. Centrifuge for 2 min in a microcentrifuge at 1,000 xg to remove the remaining packing buffer. Discard the tube.
    a. Note: the speed is important to ensure proper performance of the columns.
3. Place the column in a clean 1.5 ml microcentrifuge tube (supplied with the kit). Carefully apply the sample (10-75 µl) directly onto the top center of the gel bed.

Do not disturb the gel bed. Application of more or less than the recommended samples volume may decrease column performance.

4. After loading the sample, centrifuge the column for 4 min at 1,000 xg.
5. Keep the eluted sample at -20°C in a new Eppendorf tube (the probe will be eluted in buffer SSC).

1.8. Calculations of probe mixture concentration

1. Use 1 µl to determine the concentration and the labeling efficiency. Use Nanodrop.
   a. Use a blank of SSC buffer (used for the probes elution in the cleaning kit).
      i. SSC buffer preparation: 3M NaCl + 0.3M Sodium citrate. Autoclave. Final volume: 1l
2. Assess the absorbance of nucleic acids at a 260nm (A_{260}) and the Alexa594 at 594nm (A_{594}).

Use the program "Microarray" of Nanodrop and the option DNA-50 (i.e. dsDNA).

1.9. Calculations of labeling efficiency

To estimate how many Alexa molecules are for each 300bp (i.e. 1 probe):

$$\text{Base:dye} = (A_{base} \cdot \varepsilon_{dye}) / (A_{dye} \cdot \varepsilon_{base})$$
$$\varepsilon_{dye} = 80400\ cm^{-1}\ M^{-1}$$
$$\varepsilon_{base} = 6600\ cm^{-1}\ M^{-1}$$
$$A = \text{absorbance}$$

300pb / Base:dye → #number of Alexa/probe

Note: optimal values are over 6 Alexas/probe.

2. Catalyzed Reporter Deposition fluorescent in situ hybridization of host cells

2.1. Sample fixation

Samples are fixed with formaldehyde 37% (FA) (1 ml FA / 9 ml sample) during 15 min at room temperature, and filtered through a 0.4 µm or 0.2 µm white 25mm polycarbonate filter. Filters are kept at -20 ºC for short periods of time or at -80 ºC for long-term storage.

2.2. Attachment

This step is performed to avoid losing cells during the hybridization procedure.
Prepare 0.1% (w/w) of low gelling point agarose in MQ water.

- Boil the agarose in a microwave.
- Pour the agarose into a small petri dish and let it cool down to 40-35 ºC.
- Dip each filter quickly in the agarose (covering well both sides of the filter) and shake it to remove the excess of agarose.
- Place the filters face up onto parafilm on a glass plate.
- Let the filters dry in an oven at 37 ºC until they look white (approx. 10-30 min).
- Pipet ethanol (96-80% [v/v]) onto the filters and carefully peel them off.

2.3. Removal of chlorophyll and peroxidases

If host cells are photosynthetic the chlorophyll will interfere with Alexa594, so it needs to be removed. It is also important to inactivate endogenous peroxidases that might interfere with the CARDFISH.

To remove chlorophyll and peroxidases immerse sequentially the filters 1h in ethanol, 1h in methanol and 10min in HCl at 0.01 M, and then wash them with MQ.

2.4. Hybridization

- Prepare a humid chamber with a piece of paper. Wet the paper with ~700 µl of hybridization buffer (HB) (with the corresponding % of formamide). See below for the corresponding recipe.
    - Use a different chamber if several formamide concentrations and/or probes are used.
- Cut the filters in triangles and label the upper face of the filter by a number or any meaningful annotation.
- Prepare the hybridization mix: (18 µl HB + 2 µl host probe) * #number of filters.
- Place the filters face up on slides and completely cover them with 20 µl of the hybridization mixture.
- Place the slides in the hybridization chamber and incubate overnight at 35 ºC.

2.5. Washing

After the overnight period remove the excess of probes with the following washing buffer:

- In a falcon tube with pre-warmed (at 37 ºC) MQ water add (to 50 ml final volume):
    - 1 ml Tris 1M
    - 500 µl EDTA 0.5M
    - 50 µl 10% SDS
    - X µl NaCl 5M (see table below for the amount of NaCl to be added)
- Add MQ up to 50 ml, mix and pour half of the volume (25 ml) to an empty pre-warmed at 37 ºC falcon. Thus, it will result in two falcons with 25 ml of washing buffer.
- Put the hybridized filters in the one of the falcon tubes with washing buffer, and incubate for 10min at 37 ºC.

- Repeat the procedure in the second falcon tube.
- Equilibrate the filters with PBS for 15 min in the dark and at room temperature.

Amount of NaCl to add to the washing buffer depending on the % of formamide in the hybridization buffer.

% formamide	NaCl M	NaCl (µl) for 50 ml
20	0.145	1450
25	0.105	1050
30	0.074	740
35	0.052	520
40	0.037	370
45	0.026	260
50	0019	190
55	0.013	130
60	0.009	90
65	0.008	80
70	0.005	50

2.6. CARD-FISH reaction

Prepare the fluorochrome solution:

- First prepare the H_2O_2 stock: 200 µl PBS + 1 µl H_2O_2 (30%)
  - Vortex well the mix.
- To 1 ml of Amplification buffer (AB, see recipe below) add 10 µl of H_2O_2 stock and 4 µl of Alexa488.
  - Note: do NOT vortex the mix. Homogenize with the pipette.
- Transfer the filter to the AB mixture. Immerse well the filters in it. Incubate 1h at 46 ºC.

2.7. Washing

Wash the filters to remove the excess of Alexa488 and stop the enzymatic reaction.

- 2x10 min at room temperature with PBS.
- Wash the filters with MQ for 30-60s and let them dry.

3. VirusFISH

In this section we explain the hybridization of viruses infecting the host and free viruses.

3.1. Sample fixation

For the host-virus interaction, proceed with the same triangles already hybridized with CARD-FISH for the host. For free virus hybridization, 900 µl of samples are fixed with 100 µl of freshly filtered formaldehyde (3.7% final concentration) for 15 min at room temperature. Then, 500 µl of fixed sample are filtered through 0.02 µm pore size anodisc filters (Whatman®) (after a 0.2 µm pore size prefiltration to remove cells and debris).

3.2. Hybridization

1. Prepare a humid chamber with a piece of paper. Wet the paper with ~1 ml of MQ+Formamide (with the corresponding % of formamide).
    a. Use a different chamber if several formamide concentrations and/or probes are used.
    b. The % of formamide depends on the % GC selected during the primers designing step. If it is in a range of 40-60% GC the resulting % formamide should be 40%.
2. Prepare the hybridization mixture (adding the viral probes to the viral hybridization buffer, see recipe below)*:
    a. Keep the hybridization mixture in the dark.
    b. Vortex well.
3. Place the filters face up on slides and put on them 25 µl of the hybridization mixture.
4. Denature the viruses for 40 minutes at 85 ºC in a hybridization oven.
5. Transfer the hybridization chamber to a hybridization oven at 46 ºC and incubate for 2h.

*The final hybridization buffer volume is: **25µl HB · # number filters**

*For the viral probe we need a final concentration of 62 pg µl^{-1}/probe. Calculate the volume of probe we have to add to the HB buffer:

x = (25 µl hybrid. buffer · # Nb filters) · (62 pg/µl probe · # Nb probes) / ([viral probe concentration in ng/µl] · 1000)
x = µl of the probe mix to add to the HB

3.3. Washing

Transfer the filters to the following washing buffer and incubate for 15 min at 48 ºC.

- Washing buffer:
    - In a pre-warmed at 48 ºC falcon with MQ add (to 50 ml final volume):
        - 1 ml Tris 1M
        - 500 µl EDTA 0.5M
        - 50 µl 10% SDS
        - X µl NaCl 5M (the volume depend on the % formamide. See table below).
        - Add MQ up to 50 ml

*% Formamide in the hybridization buffer	20	25	30	35	40	45	50
µl of NaCl in 50 ml	2250	1590	1120	800	560	400	280

Finally, wash the filters with MQ to remove the washing buffer.

3.4. SYBR Gold staining

For detecting free viral particles do this SYBR Gold staining step on the hybridized anodisc filters:

- Dilute the SYBR Gold stock (10,000X) to 1,000X and keep the aliquots at -20 ºC.
- Place 97.5 µl-drops of autoclaved MQ water onto petri dishes (one drop for each hybridized anodisc piece).
- Add to each MQ drop 2.5 µl of diluted SYBR Gold (final concentration 25X). Note: resuspend the SYBR Gold in the drop for a good homogenization.
- Place the anodisc triangles (faced-up) on top of the drops and let them sit for 12 min to facilitate the SYBR Gold penetration into the capsids.
- Finally, wash the filters with MQ to remove the stain excess and let them dry.

4. <u>Microscopy</u>

4.1. Mounting of the samples on slides:

For 0.2 µm white polycarbonate filters:

1. Prepare the DAPI solution with antifading reagent (77% glycerol, 15% VECTASHIELD and 8% 20x PBS).
    a. 5 µl DAPI 0.5 mg/ml + 500 µl antifading reagent.
2. Extend the DAPI + Antifading reagent onto a slide.
3. Place the filters face up on the oil.
4. Cover the filters with a coverslip.
5. Remove residual bubbles that appear over the filters.

For 0.02 µm anodisc filters:

1. Place the anodisc filters on the slides.
2. Put a drop of a CitiFluor™ Glycerol-PBS Solution AF1 over each filter.
3. Cover the filters with a coverslip.
    a. Note: press the coverslip to expand the CitiFluor over the filters but not too much to avoid breaking the anodisc filters and introduce air bubbles.

4.2. Observation

Hosts (A_{488}): blue light (475/30 nm excitation, 527/54 BP emission and FT 495 beam splitter).

Viruses (A_{594}): orange light (585/35 nm excitation, 615 LP emission and FT 570 beam splitter).

Buffers preparation

Hybridization buffer for CARD-FISH:

- X% of deionized formamide.
- 0.9 M NaCl.
- 20 mM Tris.
- 0.01%SDS.
- 2% Blocking agent.

For 10 ml final (it can be stored in aliquots of 1 ml at –20°C) :

- X ml H_2O steril
- Y ml formamide (see table below)
- 1.8 ml NaCl 5M
- 200 µl Tris 1M pH 7.5
- 10 µl 10% SDS
- 2 ml Blocking agent 10X (Boehringer Mannheim)

(ml)	10%	20%	30%	40%	50%	60%
Formamide	1	2	3	4	5	6
MilliQ	5	4	3	2	1	---

Amplification buffer for CARD-FISH:

For 50 ml:

- 2 ml PBS 20X.
- 0.4 ml Blocking reagent (Roche, Bazel, Switzerland).
- 16 ml NaCl 5M.
- MilliQ water up to 40 ml.
- Add 4 gr of Dextran Sulfate. Heat to 40-60°C, shake until the DS dissolved completely.

Store at 4 ºC in aliquots of 1 ml for several weeks.

Hybridization buffer (HB) for VirusFISH:

For 10 ml of a 30% formamide concentration HB:

- 3 ml formamide.
- 2.5 ml SSC 20X.
- 100 µl SDS (10%).
- 400 µl EDTA (0.5M).
- 250 µl Sheared Salmon Sperm (10mg/ml).

- 250 µl Yeast RNA (10mg/ml).
- 1 ml Blocking reagent (10%).
- 1.6 ml MQ water.
- Add 2 gr of Dextran Sulfate. Heat to 40-60°C, shake until the DS dissolved completely.

Note: the last component to add is the SDS as it precipitates when there is little volume. When the buffer is prepared, filter it through 0.2 µm. Keep it at -20 ºC.
